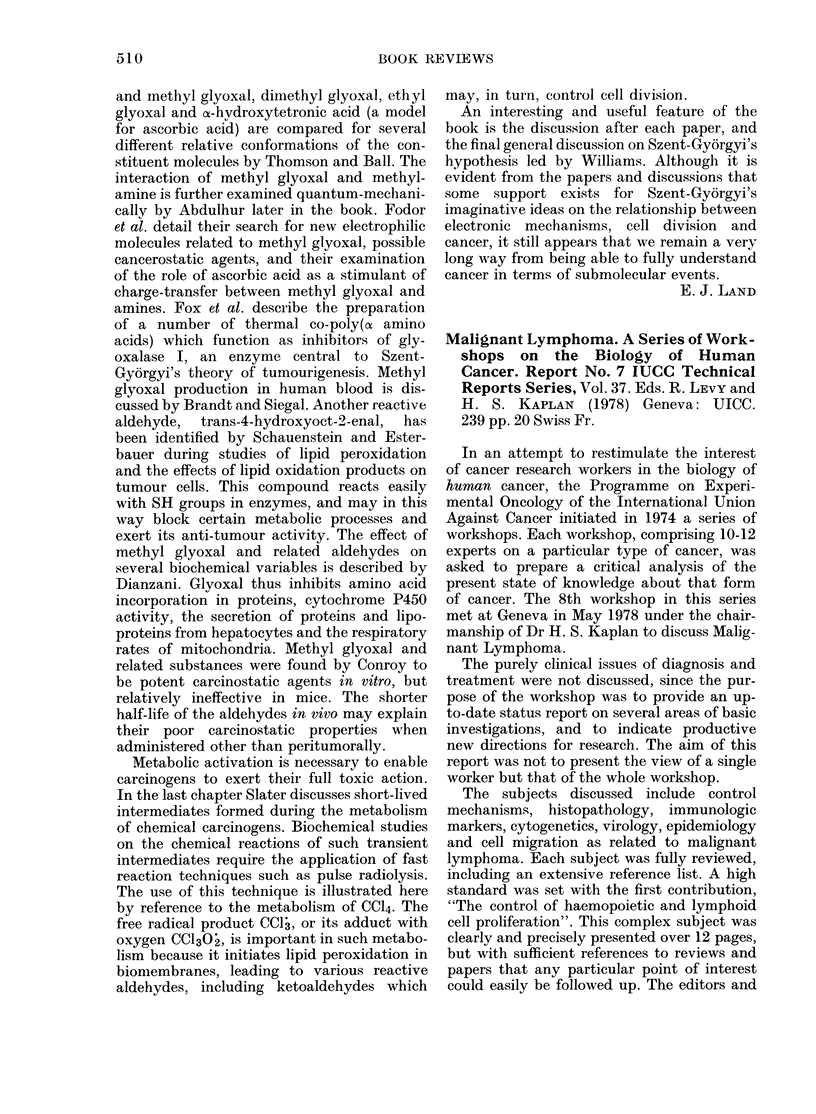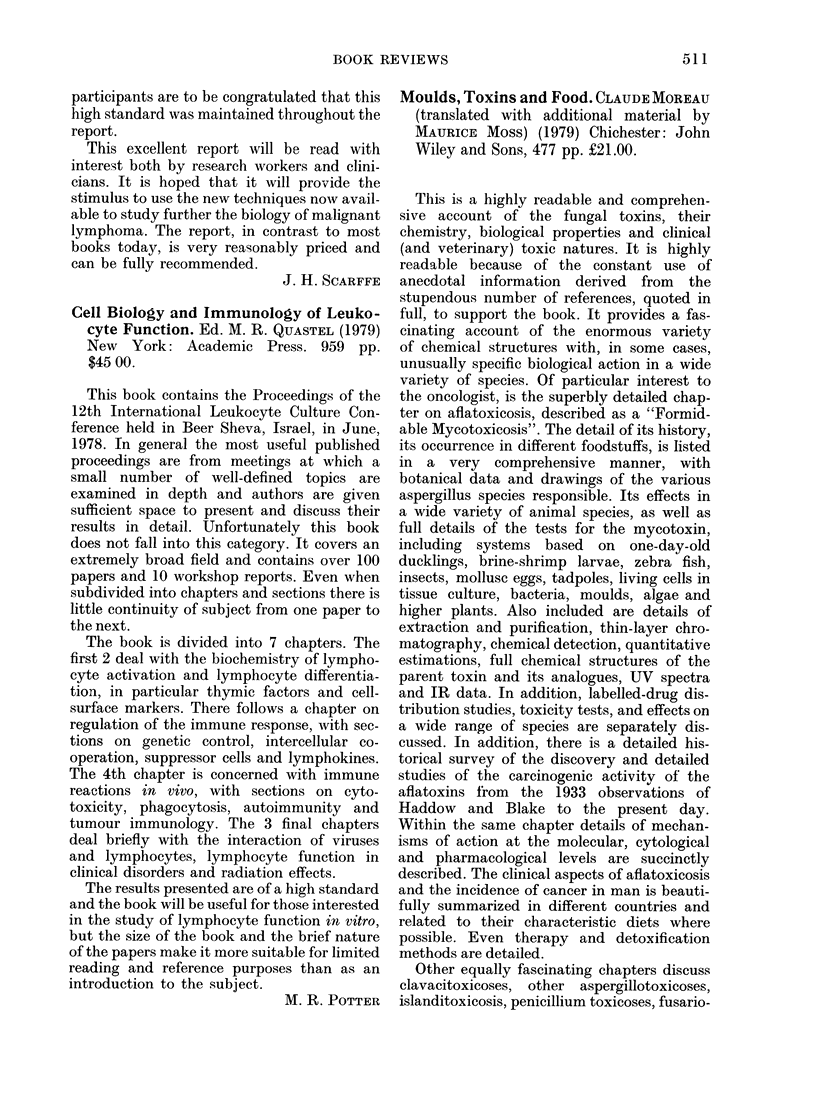# Malignant Lymphoma. A Series of Workshops on the Biology of Human Cancer. Report No. 7 IUCC Technical Reports Series

**Published:** 1980-03

**Authors:** J. H. Scarffe


**Malignant Lymphoma. A Series of Workshops on the Biology of Human Cancer. Report No. 7 IUCC Technical Reports Series,** Vol. 37. Eds. R. LEVY and H. S. KAPLAN (1978) Geneva: UICC. 239 pp. 20 Swiss Fr.

In an attempt to restimulate the interest of cancer research workers in the biology of *human* cancer, the Programme on Experimental Oncology of the International Union Against Cancer initiated in 1974 a series of workshops. Each workshop, comprising 10-12 experts on a particular type of cancer, was asked to prepare a critical analysis of the present state of knowledge about that form of cancer. The 8th workshop in this series met at Geneva in May 1978 under the chairmanship of Dr H. S. Kaplan to discuss Malignant Lymphoma.

The purely clinical issues of diagnosis and treatment were not discussed, since the purpose of the workshop was to provide an up-to-date status report on several areas of basic investigations, and to indicate productive new directions for research. The aim of this report was not to present the view of a single worker but that of the whole workshop.

The subjects discussed include control mechanisms, histopathology, immunologic markers, cytogenetics, virology, epidemiology and cell migration as related to malignant lymphoma. Each subject was fully reviewed, including an extensive reference list. A high standard was set with the first contribution, "The control of haemopoietic and lymphoid cell proliferation". This complex subject was clearly and precisely presented over 12 pages, but with sufficient references to reviews and papers that any particular point of interest could easily be followed up. The editors and participants are to be congratulated that this high standard was maintained throughout the report.

This excellent report will be read with interest both by research workers and clinicians. It is hoped that it will provide the stimulus to use the new techniques now available to study further the biology of malignant lymphoma. The report, in contrast to most books today, is very reasonably priced and can be fully recommended.

J. H. SCARFFE